# ESR Essentials: imaging of common paediatric pulmonary diseases—practice recommendations by the European Society of Paediatric Radiology

**DOI:** 10.1007/s00330-024-11268-4

**Published:** 2025-01-29

**Authors:** Jovan Lovrenski, Maria Raissaki, Domen Plut, Efthymia Alexopoulou, Süreyya Burcu Görkem, H. Nursun Ozcan, Julia Geiger, Daniel Gräfe, Chiara Sileo, Pablo Caro-Dominguez, Pierluigi Ciet

**Affiliations:** 1 Faculty of Medicine, University of Novi Sad, Institute for Children and Adolescents Health Care of Vojvodina, Novi Sad, Serbia; 2 Department of Radiology, University Hospital of Heraklion, Medical School University of Crete, Heraklion, Greece; 3 Department of Paediatric Radiology, Clinical Radiology Institute, University Medical Centre Ljubljana, Ljubljana, Slovenia; 4 Faculty of Medicine, University of Ljubljana, Ljubljana, Slovenia; 5 2nd Department of Radiology, National and Kapodistrian University of Athens, University General Hospital “Attikon”, Athens, Greece; 6 Ministry of Health Adana City Training and Research Hospital Pediatric Radiology Clinic, Adana, Turkey; 7 Department of Radiology, Subdivision of Paediatric Radiology, Hacettepe University School of Medicine, Ankara, Turkey; 8 Department of Diagnostic Imaging, University Children’s Hospital Zurich, Zurich, Switzerland; 9 Children’s Research Center, University Children’s Hospital Zurich, Zurich, Switzerland; 10 Department of Paediatric Radiology, University Hospital, Leipzig, Germany; 11 Radiology Unit, Armand Trousseau Hospital, APHP Sorbonne University, Paris, France; 12 Paediatric Radiology Unit, Radiology Department, Hospital Universitario Virgen del Rocío, Sevilla, Spain; 13 Departments of Radiology and Nuclear Medicine, Erasmus MC - Sophia Children’s Hospital, Rotterdam, the Netherlands; 14 Department of Radiology, University of Cagliari, Cagliari, Italy

**Keywords:** Lung, Infant, Paediatrics, Congenital, Diagnostic imaging

## Abstract

**Abstract:**

Chest imaging in children presents unique challenges due to varying requirements across age groups. For chest radiographs, achieving optimal images often involves careful positioning and immobilisation techniques. Antero-posterior projections are easier to obtain in younger children, while lateral decubitus radiographs are sometimes used when expiratory images are difficult to obtain and for free air exclusion. Chest CT protocols should be age-dependent to minimise radiation exposure and motion artefacts. MRI is primarily used in specialised centres to reduce radiation exposure, requiring specific expertise and sedation in younger children. Respiratory distress syndrome is a leading cause of morbidity in preterm neonates, diagnosed through characteristic radiographic findings and a history of prematurity. Bronchopulmonary dysplasia is the most common complication of extreme preterm birth and chronic oxygen therapy; imaging is used for predicting outcomes for the assessment of severe cases. Transient tachypnoea of the newborn and meconium aspiration syndrome are common in term infants, with specific imaging characteristics aiding in their differentiation. Congenital lung malformations present diagnostic and management challenges, with imaging playing a crucial role in diagnosis and surgical planning. Finally, imaging is essential in detecting complications from pneumonia in children, such as empyema and necrotic pneumonia, or in identifying foreign object aspiration.

**Clinical relevance statement:**

This review summarises current radiology practice of paediatric chest pathologies, aiding in the accurate diagnosis and management of neonatal and congenital pulmonary conditions and pneumonia complications, ultimately improving patient outcomes through precise imaging interpretation and targeted clinical intervention.

**Key Points:**

- *Chest radiographs should be systematically assessed for pathology.*
- *Ensure accurate differential diagnosis of neonatal lung diseases by collecting information on gestational age, method of delivery, presenting symptoms, ventilation type, and fetal ultrasound findings.*
- *Radiographs and ultrasound are initial diagnostic tools for paediatric pulmonary disease; CT should be reserved for complex cases. Referral to paediatric hospital should be considered when the use of chest MRI is indicated.*

## Key Recommendations

- Systematically assess paediatric chest radiographs for:
  - Lung volume
  - Focal lung abnormalities
  - Mediastinal contours, lines and shift
  - Cardiac silhouette and pulmonary vasculature
  - Configuration and position of central airways
  - Presence of pneumothorax, pneumomediastinum, or pleural effusion
  - Position of lines and tubes, if present

(Level of evidence: High, standard of care)

- Ensure accurate differential diagnosis of neonatal lung diseases by collecting:
  - Gestational age at birth and timing of imaging
  - Method of delivery
  - Presenting symptoms (e.g., staining in amniotic fluid)
  - Ventilation type, tube outputs (e.g., blood)
  - Fetal ultrasound findings suggesting congenital lesions

(Level of evidence: High, standard of care)

- Use chest radiograph and lung ultrasound as the initial diagnostic tools for paediatric pulmonary diseases. Reserve chest CT for complex cases, while chest MRI is suitable for chest wall and mediastinal lesions, repeated imaging to minimise radiation exposure, and gathering functional information, such as perfusion, ventilation and dynamic studies of central airways and diaphragm.

(Level of evidence: moderate)

## Introduction

In the complex field of paediatric pulmonary diseases, understanding imaging findings is crucial for accurate diagnosis and management. This review offers a comprehensive overview of both non-congenital and congenital pulmonary diseases, as well as miscellaneous conditions frequently encountered in clinical practice. Information is tailored for general radiologists, who may not have extensive experience in paediatric chest imaging, providing key clinical and imaging features in various imaging modalities for a confident diagnosis. Topics covered range from common conditions like pneumonia and respiratory distress syndrome of premature infants to less frequently seen pathology such as congenital pulmonary abnormalities, providing a differential diagnosis based on patient history. Essential knowledge aims to enable general radiologists to interpret paediatric chest imaging studies with confidence and contribute to the timely diagnosis and effective management of pulmonary diseases across all age groups.

## Imaging modalities

Chest imaging in children presents unique challenges, particularly in obtaining high-quality diagnostic images across different age groups [1]. For chest radiographs (CR), achieving optimal image quality often hinges on appropriate positioning, effective immobilisation, or distraction techniques. In younger children, an antero-posterior (AP) projection is typically easier to obtain compared to a posterior-anterior (PA) projection [1]. For detecting overinflation, acquiring expiratory images in this age group can be challenging. As a result, a lateral decubitus AP radiograph serves as a possible alternative, though it is not frequently used. With this technique, the lung in contact with the table is in expiration, while the lung on the opposite side is in inspiration [1]. It may also help to depict subtle pneumothorax.

For chest computed tomography (CT), protocols must be adjusted according to the patient’s age [2, 3]. For children under 5 years, high-pitch, fast CT protocols are preferred to minimise motion artefacts and reduce radiation exposure [2, 3]. In older children, proper training and coaching can enable them to perform both inspiratory and expiratory manoeuvres, which significantly enhance image quality and diagnostic accuracy. These manoeuvres can be facilitated using a spirometer, allowing inspiratory scans at lung volumes near total lung capacity and expiratory scans at volumes close to residual capacity. Achieving adequate inspiratory lung volume is critical for accurate interpretation of lung structures, such as airway dimensions, while full expiratory scans are essential for detecting airway obstructions, particularly in cases such as foreign body aspiration and small airway diseases [2–4].

Chest MRI is usually reserved for specialised centres with expertise in this modality [5, 6]. Over the past decade, the use of chest MRI has expanded to reduce the cumulative radiation dose from serial imaging and to provide dynamic assessments of central airways, diaphragmatic motion, and ventilation and perfusion [6]. Age-dependent protocols are crucial for chest MRI. In non-cooperative children, free-breathing, respiratory-triggered protocols are preferred to capture high-quality images without requiring active patient cooperation [6]. For children aged 6 months to 6 years, moderate sedation or anaesthesia may be necessary to mitigate the challenges posed by lengthy scan sessions and to ensure optimal imaging [7]. Table 1 summarises the key technical requirements for CR, CT, and MRI in paediatric chest imaging.

**Table 1:** Key technical requirements for paediatric chest imaging

Imaging modality	Key technical requirements	Recommendations for general radiologists and radiographers
Chest radiograph	• Use child age specific exposure settings • Ensure accurate positioning • Centering point: 7th thoracic vertebra • Collimation: • Superiorly: 3rd cervical vertebrae • Inferiorly: thoracolumbar junction • Laterally: skin margins of chest wall	• Minimise motion artifacts • Prepare the room beforehand • Ensure appropriate inspiration and no motion using specialised communication techniques (i.e., “take a deep breath like when you want to dive”) • Check inspiratory level by using posterior rib counts (at least eight ribs above the diaphragm) or anterior rib counts (five anterior ribs above hemidiaphragm) • Check rotation by assessing symmetry of clavicles’ size and equidistant anterior ribs
Chest CT	• Use dedicated low-dose protocols • In non-cooperative children (age < 5 years) perform ultrafast CT scan using high pitch and maximum rotation speed • In small infants and children consider the use of vacuum mattresses to reduce motion • In cooperative children, consider optimal training to perform inspiratory and expiratory manoeuvre	• Implement child-specific protocol according to dose-reduction techniques • Consider referring to a paediatric hospital for complex cases
Chest MRI	• Utilise free-breathing T2-weighted sequences for non-cooperative patients (i.e., PROPELLER/BLADE) • Use preferably 1.5-T scanner or lower for a better SNR of the lung parenchyma • Use MRI coils with close fitting to the chest according to chest size	• Consider referral to specialised paediatric centres if chest MRI is indicated but expertise in both the acquisition and interpretation of paediatric chest MRI is lacking • Consider the use of MRI in collaborative children for: • Detailed soft tissue evaluation • Mediastinum and chest wall assessment • Minimise cumulative dose in case of serial imaging • Dynamic assessment of central airways and diaphragm • Perfusion and ventilation assessment

*BLADE* Brand name SIEMENS for Turbo Spin Echo T2-weighted sequence, *CT* computed tomography, *MRI* magnetic resonance imaging, *PROPELLER* Periodically Rotated Overlapping ParallEL Lines with Enhanced Reconstruction (brand name General Electrics), *SNR* signal-to-noise ratio, *T* Tesla

## Non-congenital/medical pulmonary diseases

### Respiratory distress syndrome

Respiratory distress syndrome (RDS) is the leading cause of morbidity and mortality in preterm neonates born before 36 weeks of gestation [8]. Surfactant, produced between 24 and 34 weeks of gestation, reduces alveolar surface tension, facilitating lung expansion during inspiration [8]. Surfactant deficiency causes alveolar collapse and hyaline membrane formation, manifesting tachypnoea, intercostal retractions, and nasal flaring, necessitating mechanical ventilation [8].

**Fig. 1:**
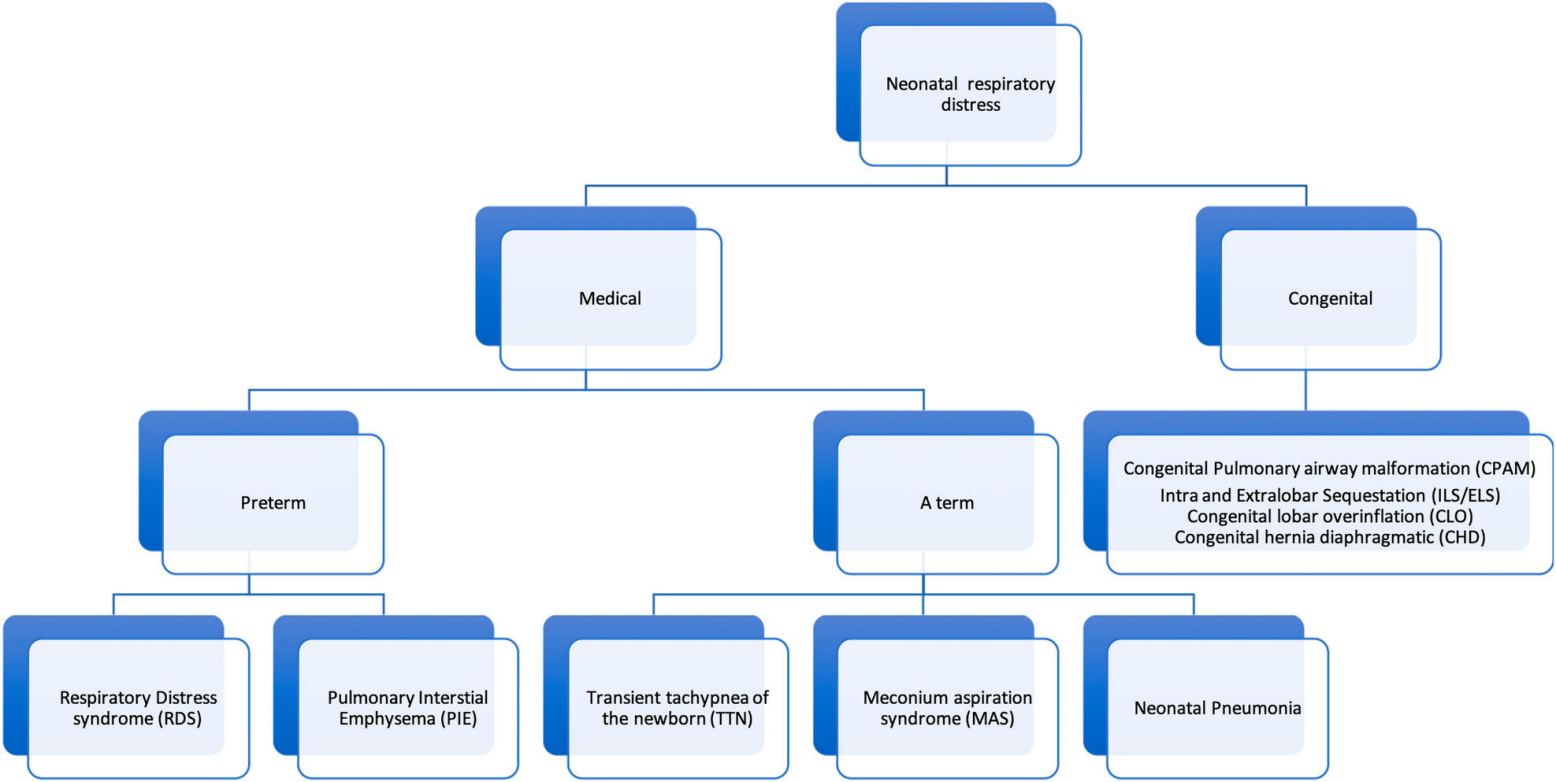
Flowchart of differential diagnosis in neonatal pulmonary disease (excluding systemic/extrathoracic manifestations, such as sepsis, severe anaemia and skeletal dysplasia) causing respiratory distress

**Fig. 2:**
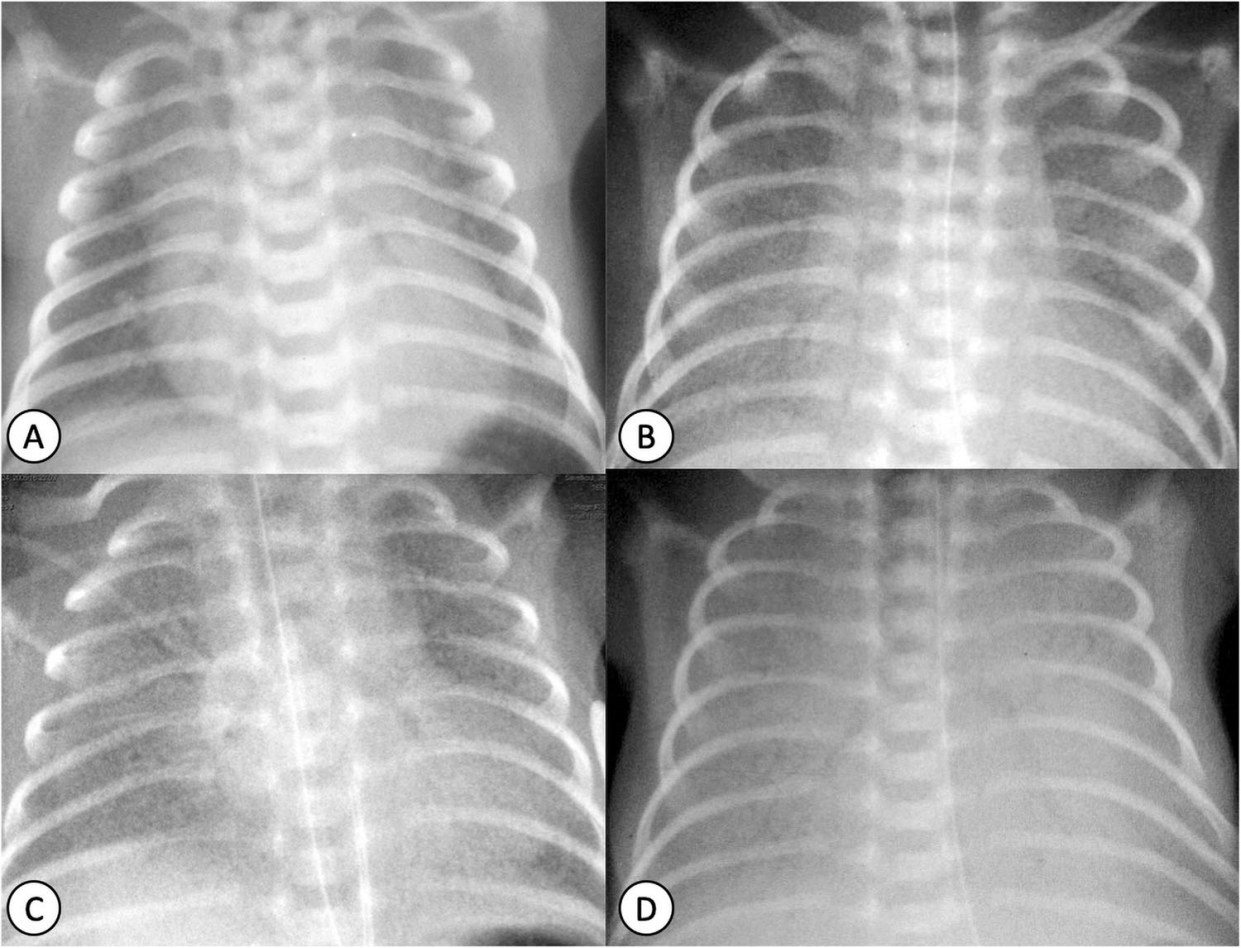
Respiratory distress syndrome grading on chest radiographs. **A** RDS grade 1: a ground-glass appearance of the lungs with well-expanded lung and with limited air bronchogram, (**B**) RDS grade 2: more prominent opacification of the lung, which is relatively well-expanded, but with increased presence of air bronchogram and still well distinct heart and diaphragmatic contours. **C** RDS grade 3: coarse granular opacification of the lungs and less distinct cardiac and diaphragmatic silhouette, widespread air bronchograms and poorly expanded lungs and (**D**) RDS grade 4: total collapse of the lungs with complete opacification, indistinct cardiac and diaphragmatic silhouettes and widespread distinct air bronchograms

Imaging diagnosis of RDS relies on a history of prematurity and characteristic radiographic findings (Table 2) [9] which allow differentiation from other common neonatal pulmonary diseases (Fig. 1). These range from hypoinflation, diffuse granular opacities to “white lungs” with air-bronchograms and bilateral silhouette sign (grades 1 to 4, Fig. 2). The four-level grading system used by radiologists may differ from the approach used by neonatologists, who prefer describing the severity of RDS on chest radiographs as mild, moderate, or severe based on the visualisation of heart contours. Typical complications of RDS include pneumothorax and pulmonary interstitial emphysema [9]. The latter is caused by interstitial air leakage related to barotrauma from mechanical ventilation and presents on CR as rounded or tubular perihilar lucencies representing interstitial air bubbles (Table 2). Following exogenous surfactant administration, radiological improvements include increased lung ventilation and reduced opacification, although hypoinflation and granular opacities may persist [9]. Prenatal corticosteroid administration and postnatal surfactant therapy significantly enhance RDS patient survival [8, 9].

**Table 2:** Summary table of common neonatal non-congenital lung diseases, imaging techniques, and key clinical and imaging findings

Neonatal lung disease	Primary radiology techniques	Key clinical information for diagnosis	Typical pattern on CR	Characteristic imaging findings	Differential diagnosis
Respiratory distress syndrome	• CR • LUS	• Preterm neonates with respiratory distress		• Low lung volumes before mechanical ventilation • Diffuse homogeneous granular opacities • Air-bronchograms • LUS: subpleural consolidations, interstitial or alveolar-interstitial oedema seen as coalescent B-lines, pleural line abnormalities (LUS findings depend on the grade of RDS)	• Neonatal pneumonia • MAS • Genetic surfactant dysfunction disorders
Bronchopulmonary dysplasia	• CR • CT • (follow-up) • MRI • (follow-up, if available)	• Preterm neonates with chronic respiratory support (supplemental oxygen—long-term mechanical ventilation) in NICU and persistent respiratory distress		• Early findings: mild perihilar opacities and fine granularity • Later findings: coarse reticular opacities (white arrow), cystic lucencies (black arrow), atelectasis and lung hyperinflation	• Neonatal pneumonia • MAS • PIE
Transient tachypnoea of the newborn	• CR • LUS	• A term neonate, delivered by C-section or history of maternal diabetes with respiratory distress		• Normal or enlarged lung volumes • Perihilar interstitial opacities and blurred-indistinct pulmonary vessels • Small pleural effusions (e.g., in minor fissure) (arrow) • Resolution of findings and symptoms within 48–72 h • LUS: “double lung point” sign, pleural abnormalities, absence of subpleural consolidations	• Neonatal pneumonia • PIE
Meconium aspiration syndrome	• CR • LUS	• Full-term or post-term neonates with respiratory distress and history of meconium staining of amniotic fluid		• Hyperinflated lungs may be the only finding in mild cases • Coarse irregular bilateral opacities with segmental areas of atelectasis (arrows), hyperinflation and pneumonitis • Pneumothorax, pleural effusion and PIE • LUS**:** subpleural consolidation(s) with air-bronchogram, incompact or compact pattern of B lines, pleural line abnormalities, ± pleural effusion	• Neonatal pneumonia • BPD
Pulmonary interstitial emphysema	• CR	• Neonates on long-term treatment with invasive ventilation		• Perihilar rounded lucencies representing small air bubbles (arrows), ± pneumothorax and or pneumomediastinum	• RDS • BPD
Neonatal pneumonia	• CR • LUS	• Both preterm and full-term neonates, presenting with respiratory distress and/or non-respiratory symptoms • Fever is not common		• Alveolar opacities, usually diffuse and bilateral, or lobar consolidation (white arrow) with air bronchogram (black arrow) • Coarse irregular opacities and/or reticular opacities • ± Pleural effusion • LUS: subpleural consolidation with or without air/fluid-bronchogram, focal area of alveolar-interstitial oedema, ± pleural effusion	• RDS • TTN • MAS

*BPD* bronchopulmonary dysplasia, *CR* chest radiograph, *CT* computed tomography, *LUS* lung ultrasound, *MAS* meconium aspiration syndrome, *MRI* magnetic resonance imaging, *NICU* neonatal intensive care unit, *PIE* pulmonary interstitial emphysema, *RDS* respiratory distress syndrome, *TTN* transient tachypnoea of the newborn [1, 3, 15]

### Bronchopulmonary dysplasia

Bronchopulmonary dysplasia (BPD) also known as chronic lung disease of prematurity, is the most common complication of extreme preterm birth [10]. Its incidence increases with decreasing gestational age, with preterms born before 23 weeks having over 80% risk of developing BPD [10]. Clinical diagnosis of BPD applies to children born before 32 weeks who require chronic oxygen therapy at 36 weeks postmenstrual age [11].

While imaging plays a limited role in BPD diagnosis according to current guidelines [11, 12], it holds predictive value for desaturation events and hospital admissions [13, 14]. Currently, cross-sectional imaging is only recommended for BPD patients with severe respiratory symptoms and recurrent hospital admissions [12]. CT and MRI help phenotype BPD patients, revealing heterogeneous imaging patterns despite the common clinical diagnosis of severe BPD [15, 16]. Structural abnormalities quantified on chest MRI have been shown to predict clinical and lung function outcome in BPD patients [17].

**Fig. 3:**
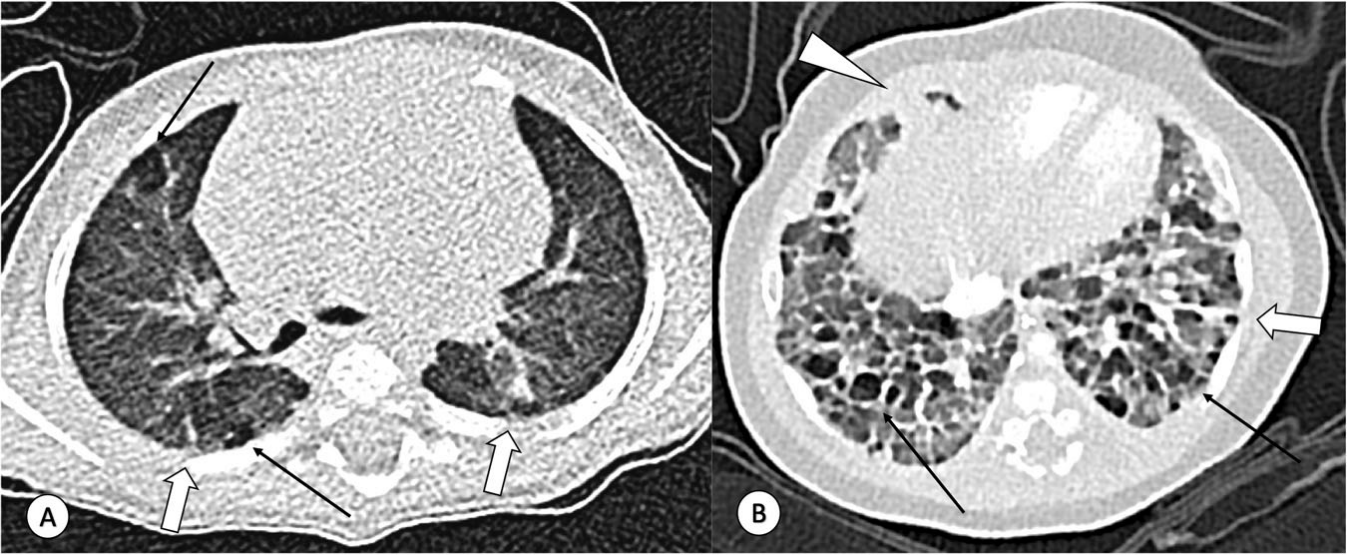
Bronchopulmonary dysplasia (BPD): **A** Free-breathing CT image of a mild BPD patient and (**B**) Photon Counting CT image of a severe BPD patient. Typical features of BPD include areas of low attenuation (arrows) observed in both mild and severe BPD cases, linear opacities (thick arrow), and consolidation (arrowhead) in severe BPD patients

Typical CR findings of BPD are shown in Table 2 [8, 9]. CT scans may be performed in tertiary centres around 6 months corrected postmenstrual gestational age, and in severe BPD cases during late adolescence [12, 14]. CT findings of BPD include linear or subpleural triangular consolidations, focal areas of low attenuation, bronchial wall thickening, and architectural distortion (Fig. 3) [14]. While chest MRI has been proposed as an alternative to CT, it has not yet been widely adopted in clinical practice [16, 18].

### Transient tachypnoea of the newborn

Transient tachypnoea of the newborn (TTN) is temporary respiratory distress seen in a term infant, due to retention of amniotic fluid in the lungs [9]. It is the commonest cause of respiratory distress in term infants, with an estimated incidence of 4–6 cases per 1000 term births [19], and is generally benign and self-limiting. Clearance of fetal lung fluids normally begins before birth and continues through delivery promoted by catecholamines and other delivery-released hormones [19]. Typical risk factors for TTN are caesarean delivery, maternal diabetes and obesity [19]. In TTN, tachypnoea develops right after birth and up to 2 h after delivery and usually resolves within 72 h. Characteristics CR findings of TTN are normal or hyperinflated lung, perihilar interstitial opacities, blurred pulmonary vessels, and small pleural effusions, as shown in Table 2 [8, 9]. TTN and RDS can appear similar radiologically, but TTN typically shows normal or hyperinflated lungs, whereas RDS presents with hypoinflation [8, 9]. Moreover, TTN occurs in term infants, while RDS is a disease of prematurity.

### Meconium aspiration syndrome

**Fig. 4:**
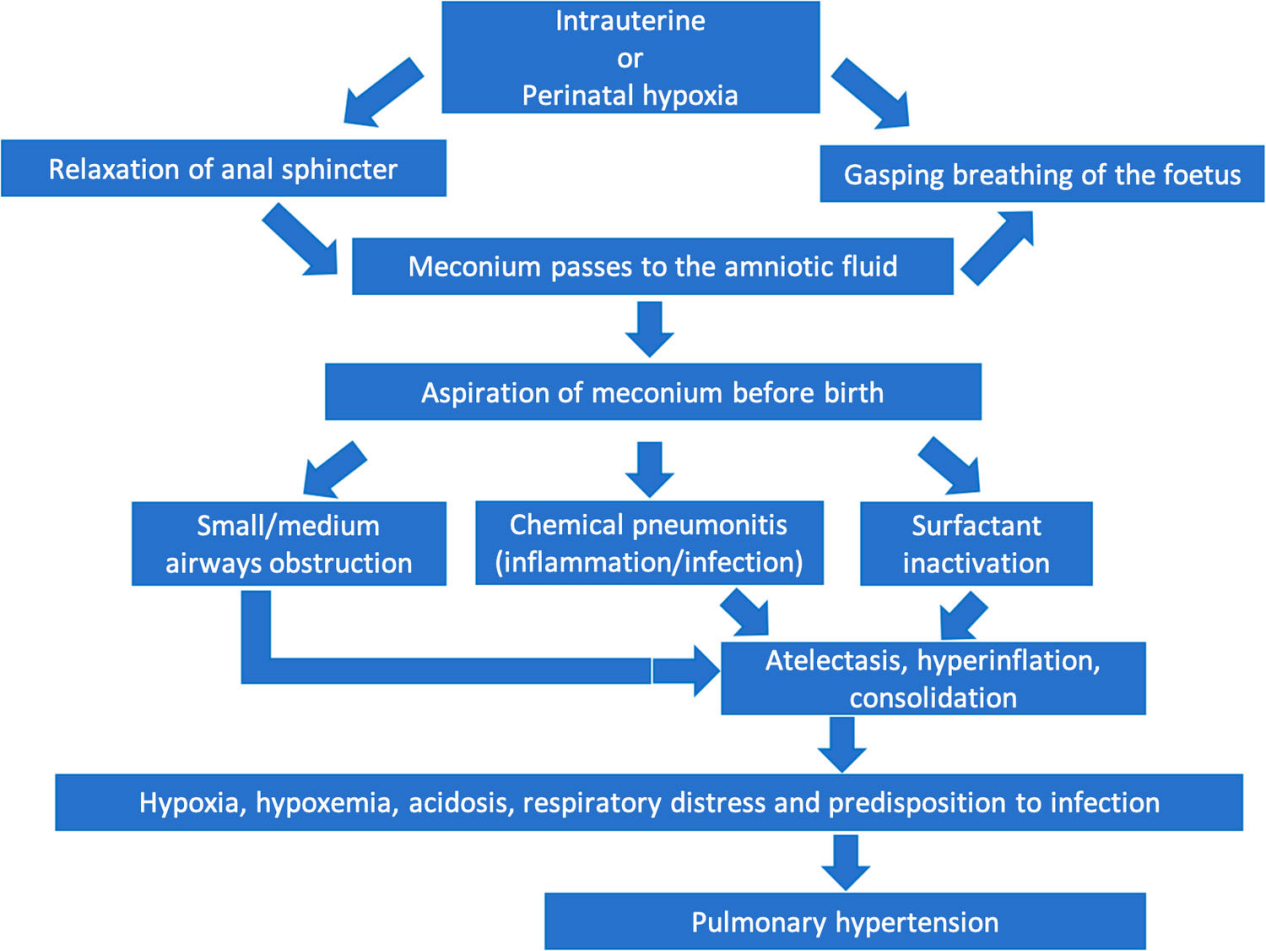
Pathophysiology of meconium aspiration syndrome (MAS): Prolonged hypoxia stimulates fetal breathing and gasping, leading to inhalation of amniotic fluid and increased peristalsis with anal sphincter relaxation. This allows meconium to pass into the amniotic fluid, which can be aspirated during fetal gasping or initial breaths. Aspirated meconium causes airway obstruction, resulting in areas of atelectasis and/or hyperinflation distally. It also triggers inflammatory pneumonitis with epithelial damage and reduces surfactant activity and synthesis, further promoting atelectasis and consolidation. Decreased alveolar ventilation and ventilation-perfusion mismatch lead to hypoxaemia, causing respiratory distress and increasing infection risk. Persistent pulmonary hypertension is an associated complication of MAS

Meconium aspiration syndrome (MAS) manifests as respiratory distress in infants who aspirate meconium-stained amniotic fluid, a sterile, black-green faecal material typically produced by fetuses after 34 weeks of gestation [20]. MAS is rarer than RDS and TTN, with a reported incidence of 0.1–0.4% of births [20]. Risk factors include delivery after 40 weeks gestational age, vaginal breech or caesarean delivery, maternal fever, and intraamniotic inflammation or infection. Pathophysiology involves airway obstruction by meconium plugs, inflammation, and infection, resulting in severe respiratory distress [20, 21] (Fig. 4). Clinical signs of MAS include evidence of perinatal asphyxia, fetal growth restriction or postmaturity [20]. Respiratory distress is usually evident at birth or within a few hours [7, 8]. CR findings of MAS are hyperinflated lungs, coarse irregular bilateral lung opacities with segmental atelectasis and pneumonitis (Table 2) [8, 9]. Imaging can help to differentiate MAS from other causes of neonatal respiratory distress, particularly pneumonia [8, 9], whereas in cases of bilateral, coarse interstitial opacities, differentiation between MAS and neonatal pneumonia is impossible and neonates receive empirical antibiotic treatment [8, 9]. Moreover, pneumonia is associated with risk factors, such as maternal infection, premature rupture of membranes, or prolonged labour, with symptoms emerging within hours to days after birth, differing from the immediate presentation seen in MAS. Pneumothorax may occur in up to 10–20% of cases [22]. Severe cases may necessitate extracorporeal membrane oxygenation, with increased risks of bleeding and stroke [20]. Cross-sectional imaging is generally unnecessary, unless unresponsive to standard therapy, to rule out alternative causes of severe respiratory distress [8, 9].

## Congenital lung malformations

Congenital lung malformations (CLMs) encompass various developmental anomalies affecting the lungs, airways, and vasculature [23]. Clinical presentations range from asymptomatic cases to severe respiratory distress or recurrent infections [23]. Improved prenatal imaging techniques (not discussed herein due to limited relevance to general radiologists), namely obstetric ultrasonography (US) and fetal MRI, contribute to the growing prenatal diagnosis of CLM [23, 24]. CR is often the initial screening modality for postnatal CLM detection, despite its relatively low sensitivity [23, 25]. Suspicion arises when persistent opacities or lucencies are observed within the same lobe in children with recurrent infections [25]. In most instances, further characterisation and surgical planning necessitate additional cross-sectional imaging, typically contrast-enhanced CT (CECT) or contrast-enhanced MRI (CEMRI) [23, 26–29].

### Congenital pulmonary airway malformation

Congenital pulmonary airway malformation (CPAM) is the most common type of CLM, with an estimated incidence of 1 per 8300 live births [23]. CPAM arises from abnormal bronchoalveolar tree development due to hamartomatous proliferation [23]. Histopathologically, CPAM is classified into five types according to the Stocker classification with the most common including: Type 1 (cysts > 2 cm) representing 50–70% of cases, Type 2 (cysts < 2 cm) accounting for 15–30% of cases, and Type 3 (microcystic < 5 mm) representing 5–10% of cases [23]. The latter can also present as a solid mass, often resulting in mediastinal shift, which complicates differentiation from pleuropulmonary blastoma (PPB), a malignant tumour associated with CPAM [23]. PPB is classified into three types: Type 1 (cystic), Type 2 (mixed cystic and solid), and Type 3 (completely solid) [23]. Differentiating Type 1 PPB from CPAM Type 4, which exhibits large cysts typically greater than 10 cm, is particularly challenging [23]. Currently, testing for somatic mutations in DICER1 is used to assess potential malignancy predisposition, with DICER1 heterozygous germline mutations present in up to 66% of PPB [23]. Additionally, CPAM is associated with other tumours, such as adenocarcinomas and squamous cell carcinoma, both linked to mucinous cell degeneration and KRAS gene mutations, one of the most frequently mutated genes in lung cancer [23].

**Fig. 5:**
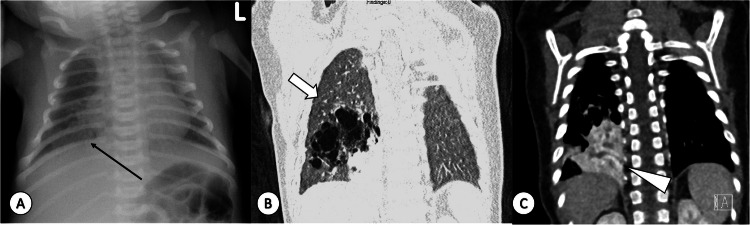
Mixed lesion comprising congenital pulmonary airway malformation (CPAM) type 2 and Intralobar sequestration (ILS) in a 1-week-old girl. **A** Antero-posterior (AP) chest radiograph revealing a poorly defined lesion in the right lung base (thin arrow in **A**). **B** CT, coronal plane, lung window, highlighting multiple cysts smaller than 2 cm associated with the CPAM type 2 component (thick arrow). **C** CECT, mediastinal window displaying a soft tissue-enhancing lung lesion in the right lower lobe (arrowhead), supplied by the descending aorta and draining through the pulmonary vein (not shown)

Imaging plays a crucial role in both diagnosis and presurgical assessment of CPAM [23, 25–29]. On CR, CPAM may appear as a focal opacity with fluid-filled cysts (Fig. 5A) [25]. Resorption of amniotic fluid alters radiographic appearances, resulting in lucent air-filled masses with or without compression of surrounding lung parenchyma and mediastinum [25]. CECT at 2 months of age is the gold standard for defining CPAM size, location, and vascular supply, aiding thoracic surgeons in surgical planning (Fig. 5B) [23, 26]. Dedicated radiology reports are available to summarise crucial CPAM features [30]. MRI for assessment of CPAM characteristics carries the advantages of evaluating vascular supply without contrast use and enabling long-term follow-up of asymptomatic children without radiation exposure [27–29]. Treatment strategies for CPAM vary among paediatric surgeons, with some advocating for early surgery in the first year of life to promote compensatory lung parenchymal growth and prevent complications from recurrent infections [23]. Those in favour of early surgery also consider the unclear risk of malignant transformation in CPAM [23]. Alternatively, a watch-and-wait strategy with long-term follow-up in asymptomatic cases may be preferred, as invasive surgery and general anaesthesia may have negative effects on long-term neurodevelopment and some potentially serious or even life-threatening complications [23]. Timing of resection is still debated; some surgeons prefer earlier surgery, by 4 months of age and some as late as 1 year of age, although delayed resection has not shown improved outcomes [23].

### Bronchopulmonary sequestration

Bronchopulmonary sequestration (BPS) is the second most common CLM, with an estimated incidence of 1 to 10 in 35,000 live births [23]. It presents as a dysplastic segment or lung lobe, not linked to the tracheobronchial system, with a blood supply from a systemic artery instead of the pulmonary artery and draining via the pulmonary or systemic veins [23]. BPS is categorised into intra- (ILS, 75% cases) and extra-lobar (ELS, 25% cases) based on arterial and venous supply and the presence of separate visceral pleura [23]. ILS appears within the visceral pleura and is supplied by one or multiple aberrant arteries originating from the descending thoracic aorta, typically branching to the lower lobe (the most common location) [23]. The venous drainage of ILS is often to the left atrium through the pulmonary veins. Conversely, ELS is covered by a distinct pleura and receives its arterial supply from the thoraco-abdominal aorta, including visceral abdominal arteries (i.e., coeliac artery) [23]. ELS venous drainage occurs through the portal vein or the systemic azygos or hemiazygos veins into the inferior vena cava and then to the right atrium. ELS is usually located in the thoracic cavity but can also develop below the diaphragm in the abdomen (subdiaphragmatic ELS) [23]. ELS often presents early in life with symptoms like respiratory distress, cyanosis, and recurrent infections, while ILS typically manifests later with recurrent infections in the same pulmonary lobe [23].

Imaging is pivotal for diagnosis and surgical planning, particularly when echocardiography indicates significant shunting with the risk of congestive heart failure [23, 26]. CR reveals nonspecific findings, including focal opacity, primarily in the lower lobes, in patients with recurrent infections [25]. Cystic lesions may be visible in cases of combined BPS and CPAM, termed “mixed/hybrid lesions” (Fig. 5C) [23, 26]. CECT determines arterial and venous supplies, location, size, and potential coexistence of CPAM [23, 26]. CEMRI as an alternative is more challenging due to anaesthesia requirement and longer scan duration, and is performed only in specialised centres [27–29].

### Congenital lobar overinflation

**Fig. 6:**
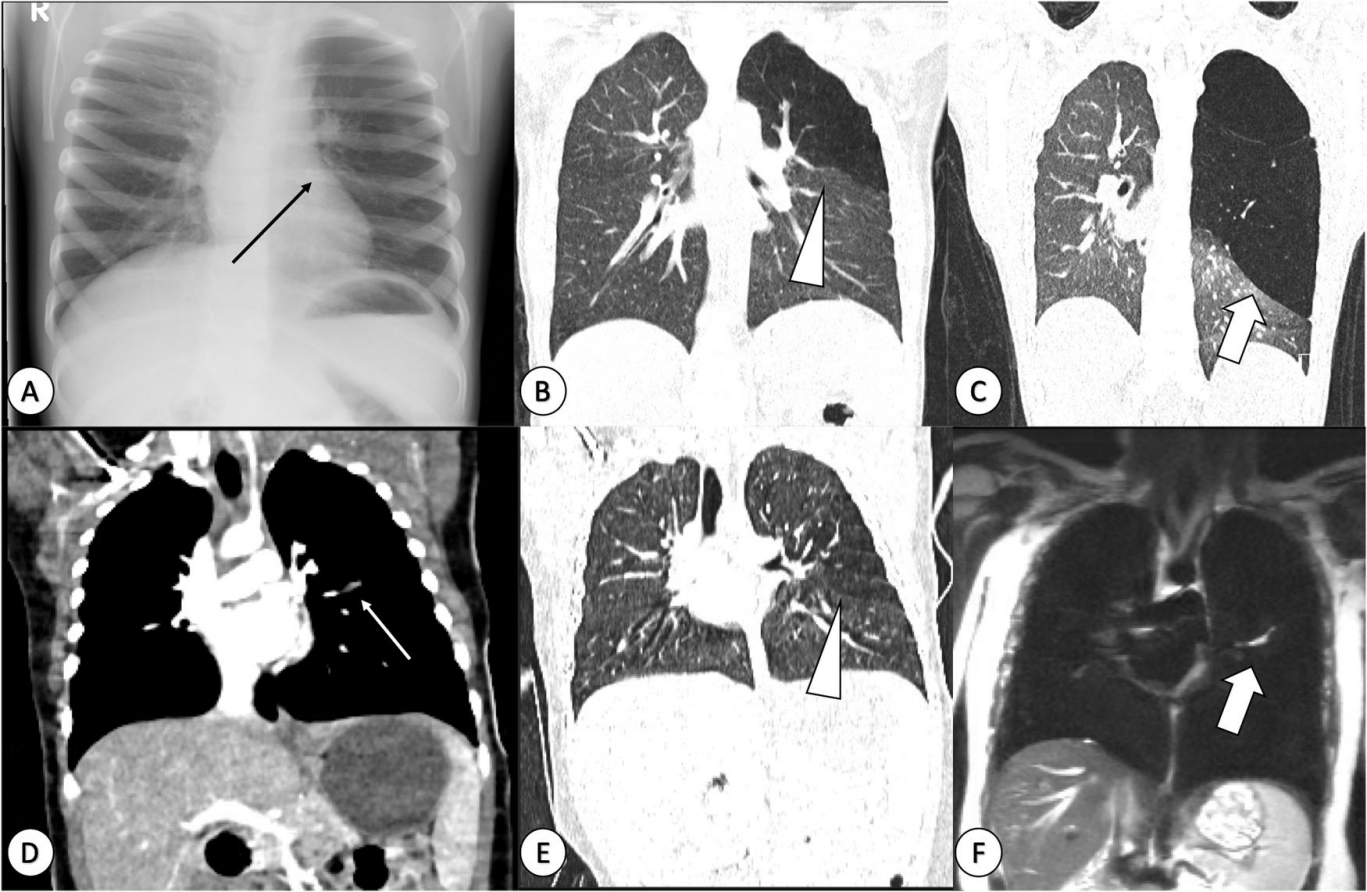
Comparison of congenital lobar overinflation (CLO) and bronchial atresia (BA) in children. **A** Chest radiograph, (**B**) coronal CT lung window image of a 3-year-old girl, and (**C**) a follow-up CT at 12 years. Notice the left upper lung lucency and asymmetric expansion compared to the right (arrow in **A**), corresponding to hyperinflated left upper lobe on the CT at the same age (arrowhead in **B**). By age 12, the patient presents with symptomatic progressive hyperinflation of the left upper lobe (thick arrow in **C**). **D** Post contrast CT, coronal plane, mediastinum window and (**E**) coronal plane, lung window and (**F**) coronal T2-weighted single-shot fast spin echo MRI in a 7-year-old boy with bronchial atresia in the apical segment of the left lower lobe. Observe the bronchocele with soft tissue density (arrow in **D**), hyperinflation of the apical segment of the lower lobe displaying lower density (arrowhead in **E**), and increased T2 signal on MRI indicating mucus within the bronchocele (thick arrow in **F**)

Congenital lobar overinflation (CLO) involves focal or segmental lung overinflation without wall destruction, often due to bronchial obstruction (ball-valve mechanism) [23, 31]. Bronchial obstruction may be caused by intrinsic factors, such as the absence of bronchial cartilage, bronchial stenosis, or bronchomalacia, or by extrinsic factors, such as a vascular ring. However, in 50% of patients, CLO is idiopathic, and a clear cause cannot be identified [23]. It most commonly affects the left upper lobe (40–45% cases), followed by the right middle (30% cases), right upper (20% cases), and lower lobes (5% cases). The historical prevalence of CLO was estimated to be approximately 1 in 20,000 to 30,000. This was during a time when first-generation CT scanners could not reliably differentiate between CLO and bronchial atresia. In recent studies, it has been shown that CLO can constitute up to 10% of CLM, with a higher incidence in males, demonstrating a male-to-female ratio of 3:1 [23, 31]. Children with CLO are typically symptomatic in the neonatal period and within the first year of life, presenting with progressive hyperinflation of the affected lobe [23]. This can lead to rapidly developing respiratory distress, often due to concurring mediastinal shift and compression of surrounding lung parenchyma (Fig. 6A–C). Diagnosis relies on imaging, which also guides surgery in symptomatic patients [23, 26, 31].

On CR, CLO typically presents as a large lucent lung segment or lobe causing compression and displacement of adjacent structures, namely the diaphragm or mediastinum (Fig. 6A–C) [25, 26]. Initial postnatal CR may show diffuse opacification with mass effect due to retained fluid, resolving in follow-up radiographs [25, 26]. CT reveals a low-attenuation region with hyperinflated lung parenchyma involving a segment or entire lobe (Fig. 6A–C) [23, 26]. MRI with conventional MR sequences to assess CLO is challenging because increased air content does not produce signal [27–29], and distinguishing CLO from CPAM on MRI can be difficult if cyst walls are not clearly visualised [27–29].

### Bronchial atresia

Bronchial atresia (BA) occurs due to focal obliteration of (sub-)segmental bronchi, leading to hyperinflation of the lung distal to the obstruction via collateral ventilation through the pores of Kohn and channels of Lambert [23, 26]. The Pores of Kohn typically become well developed by the age of 3 to 4 years, while the Channels of Lambert reach full development around 6 to 8 years of age. This developmental timeline helps explain why progressive hyperinflation of lung parenchyma surrounding the BA is more commonly observed in older children. BA carries an estimated prevalence of 1.2 cases per 100,000 live births. Despite presenting as an isolated abnormality on imaging, on pathology examination, it often coexists with other CLM, especially CPAM and BPS [23, 26, 32]. Most BA patients are asymptomatic, often diagnosed incidentally in adulthood, although recurrent infections may occur [23, 26].

Radiographically, BA presents with segmental hyperinflation like CLO, albeit usually to a lesser extent [25, 26] (Fig. 6D–F). Accumulation of mucus in the obstructed bronchus (bronchocele) may manifest as a tubular opacity (Fig. 6D–F) [25]. CT reveals segmental or lobar hyperinflated and low-attenuating lung parenchyma, with or without bronchocele (Fig. 6D–F) [26]. MRI, offering a clear depiction of the high T2 signal within the bronchocele, serves as an alternative to CT (Fig. 6D–F) in specialised centres [27]. Differential diagnosis includes CPAM, which has cystic components rather than overinflation, and CLO, which is typically larger in size and without a bronchocele [23, 26].

### Congenital diaphragmatic hernia

Congenital diaphragmatic hernias (CDH) result from defective diaphragm formation, causing abdominal structures to herniate into the chest, leading to parenchymal compression and lung hypoplasia [33]. CDH is mainly diagnosed prenatally, typically affecting the posterior hemidiaphragm (Bochdalek hernia), located predominantly on the left side (95% cases) [33]. Anterior hernias (Morgagni) are rarer (less than 5%), smaller than Bochdalek hernias, and usually not associated with lung hypoplasia [33]. Right-sided hernias exhibit more severe symptoms and poorer outcomes due to liver herniation [34]. Prenatal imaging plays an important role in CDH assessment and quantifies fetal lung volume for postnatal prognosis [24, 34].

**Fig. 7:**
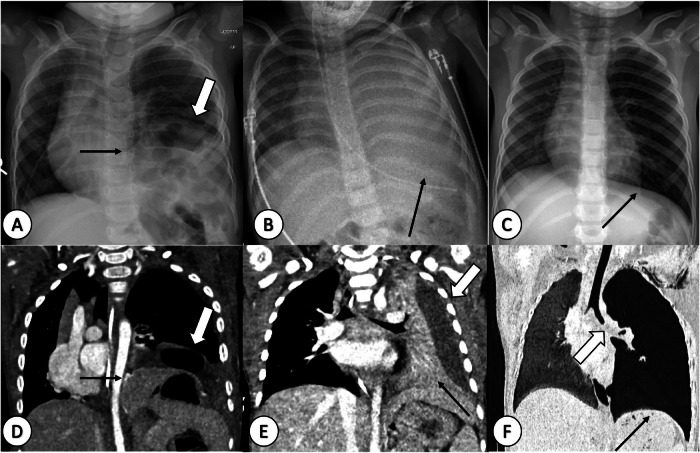
Radiographic-CT correlation. Recurrence of congenital diaphragmatic hernia (CDH) (**A**, **D**), complicated pneumonia with empyema (**B**, **E**), and foreign object aspiration (FOA) (**C**, **F**) in a 1-year-old girl, 3-year-old boy, and 2-year-old girl, respectively. **A**–**C** Anterior-posterior chest radiograph, (**D**, **E**) contrast-enhanced CT (CECT) and (**F**) coronal minimum intensity projection (MinIP). **A** and **D** reveal the surgical patch (black arrows) used for CDH correction, outlining the diaphragmatic contour and herniated bowels loop in the left hemithorax (thick arrows). **B** demonstrates complete opacification of the left lung (arrow) with loss of diaphragm and heart contours, indicative of the silhouette sign. This finding, confirmed on CECT, reveals a combination of pleural effusion (thick arrow in **E**) and atelectasis of the left lung (thin arrow in **E**). **C** depicts hyperinflation of the left lung (thin arrow), more conspicuous on CT (thin arrow in **F**) with an obstructing material in the left main bronchus (thick arrow in **F**). At bronchoscopy, a toy plastic piece and mucus plugs were extracted

Postnatal CR reveals intrathoracic air-filled bowel loops, mediastinal shift, and, in left-sided hernias, an abnormal feeding tube position when the stomach is herniated in the thorax [34]. Postnatal LUS assesses herniated contents presurgically and aids postoperative follow-up [34]. CT or MRI is usually not necessary for surgical planning. For CDH recurrence assessment, CR with two projections (AP or PA and lateral view) is recommended [34] (Fig. 7A). If CR findings are inconclusive, non-contrast T2-weighted MRI or CECT can confirm recurrence (Fig. 7D). CECT is instrumental in evaluating herniated organs and identifying potential complications, such as bowel loop incarceration, adhesions, and volvulus, which occur in approximately 20% of patients with recurrent CDH [34] (Fig. 7). In cases of recurrence, contrast is administered intravenously to assess wall enhancement in incarcerated bowel loops and exclude necrosis [35]. CECT also helps to assess possible congestion of herniated mesenteric vessels into the thorax and facilitates differentiation of herniated abdominal organs due to their different contrast enhancement [35]. Recurrence of CDH is common, often situated posterior to the surgical patch and associated with parenchymal abnormalities, like fibrotic changes and emphysema [36].

## Pneumonia and complicated pneumonia

Pneumonia is the main cause of death in children under 5 years worldwide [37]. Pathogens include bacterial, viral, or fungal microorganisms [37]. A presumptive diagnosis of the pathogens can be made based on the child’s age and whether it was a community or nosocomial pneumonia (Table 3) [38]. Clinical symptoms include cough, fever, and shortness of breath, while neonates may present with symptoms resembling other conditions like TTN, RDS, or MAS [37]. In community-acquired pneumonia (CAP), imaging is not required for the diagnosis, unless there is no response to outpatient treatment, which requires hospital admission due to suspected complications [38].

**Table 3:** Association between age and pathogen causing pneumonia in children

Age/immune status	Perinatal (up to 1 month)	Infants to young children (from 1 month to 5 years)	Children older than 5 years	Immunocompromised children
Bacterial	> 50% cases: Group B *Streptococcus* with early onset pneumonia (transvaginal infection)	Less common than viral infection Most common pathogen: *Streptococcus pneumoniae* Others: • *S. aureus* • S. pyogenes*	Most frequent pathogens: • *Streptococcus pneumoniae* • *Mycoplasma pneumoniae* • *Chlamydia pneumoniae*	Different pathogens, likely atypical or fungal infections (e.g., *Aspergillus* in bone marrow transplant or *Pseudomonas*)
Viral	Transplacental infection: TORCH group	Viral infection 80% cases, especially in children below 2 years Most frequent pathogen: Respiratory syncytial virus (RSV)		

*TORCH* toxoplasmosis, rubella, cytomegalovirus, herpes

* Higher incidence of necrosis and empyema

**Fig. 8:**
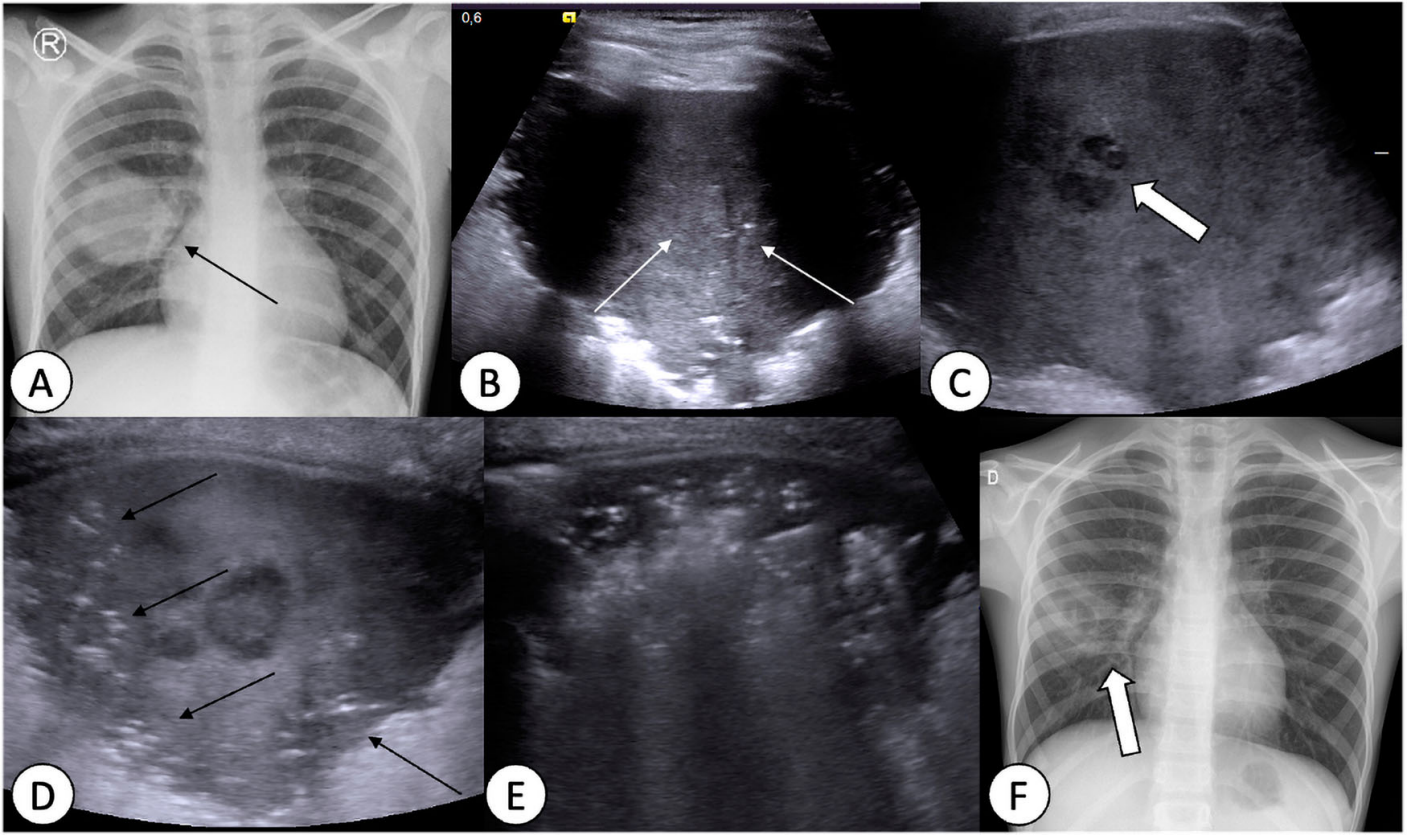
Clinical progression of round pneumonia in an 11-year-old boy. **A** Chest radiograph (CR) on admission revealing a round opacity (arrow) within the right hemithorax. **B** Lung ultrasound (LUS) shows oval-shaped subpleural consolidation with mild central increase in echogenicity (white arrows) related to the pre-necrotic stage of round pneumonia. **C** Two days later, LUS revealing distinct small hypoechogenic zones of necrosis (thick arrow). **D** Reaeration starting at the periphery of consolidation (thin black arrows), with persistent central necrosis observed after 3 days. **E** One week later, lung consolidation significantly reduced in size with diffuse reaeration and absence of detectable necrotic zones. **F** Follow-up CR confirming significant regression of pneumonia

Radiography helps in detecting nosocomial pneumonia and identifying radiological patterns. Typical CR and CT findings of lobar pneumonia, bronchopneumonia and interstitial pneumonia are shown in detail in Table 4 [37]. However, these patterns lack sensitivity and specificity for determining aetiology. A unique type of pneumonia seen only in children is round pneumonia, which presents as a round opacity on CR, typically in contact with the pleura, hilum, or pulmonary fissure [39]. This form of pneumonia is most commonly caused by bacteria, with *Streptococcus pneumoniae* responsible for about 90% of cases. Round pneumonia predominantly affects children between the ages of 5 and 12 years, as the incomplete development of collateral ventilation predisposes this age group to the condition (Fig. 8) [39].

**Table 4:** Radiological pattern of pneumonia with chest radiograph and computed tomography findings

	Lobar pneumonia	Bronchopneumonia	Interstitial
Pattern drawing			
CR findings	Lobar and/or segmental consolidation with shadowing extending to a well-defined pleural/fissural margin ± air bronchogram ± pleural effusion	Patchy consolidations with primarily basal distribution, with peribronchial involvement, often coalescing	Perihilar reticular pattern with centrally distributed peribronchial cuffing, hyperinflation and sometimes focal atelectasis
Sensitivity and Specificity of CR findings	Sensitivity 72% and specificity 51% for bacterial infection		Sensitivity 49% and specificity 72% for viral pneumonia
CT findings	Segmental or lobar consolidation with air bronchograms, pleural effusion/empyema (pleural layer enhancement), and abscesses (low-density area within the consolidation ± air with enhancing wall)	Peribronchial patchy consolidation and GGO, with mucus plugs	Diffuse or patchy area of GGO, tree-in-bud pattern (indicative of bronchiolitis), hyperinflation and mosaic attenuation
Most frequent pathogens	• 95% *Streptococcus pneumoniae*	• *Staphylococcus aureus* • *Klebsiella pneumoniae* • *Pseudomonas aeruginosa*	• Respiratory syncytial virus (RSV) • Influenza A/B • Human metapneumovirus (hMPV) • Adenovirus • Parainfluenza virus

*CR* chest radiographs, *CT* computed tomography, *GGO* ground glass opacity

LUS complements CR in pneumonia assessment (Fig. 8) [40], particularly for detecting small pleural effusions as low as 10 mL and assessing fibrinous septations [37]. LUS aids in distinguishing pleural effusions from consolidated lung and peripheral lung abscesses from empyema [37, 38]. Selection of treatment strategies depends on effusion thickness (> 2 cm) and the presence of septations on LUS, with options ranging from thoracentesis with fibrinolysis to video-assisted thoracoscopy [37, 38].

### Complications

Complications of CAP can be pulmonary or systemic, and imaging plays a crucial role in their assessment [37]. On CR, blunting of the costophrenic angle or the meniscus sign, indicate pleural effusion (Fig. 8B), while a convex border to the lung parenchyma suggests a loculated effusion [37]. Air-filled cavities or air-fluid levels suggest cavitary necrosis, lung abscess, or empyema. CT is used in refractory cases or recurrent infections to investigate predisposing factors or underlying malignancies [37]. LUS in the hands of experienced operators, or MRI in cooperative children are valid alternatives to CT. In necrotic pneumonia, CECT shows hypodense non-enhancing lung parenchyma and MRI indicates areas of decreased signal on T1-weighted images and increased signal on T2-weighted images [37]. Moreover, CT precisely locates and measures effusions (Fig. 7E) but may miss septations in empyema, unlike MRI, which visualises septations on T2-weighted images [37]. Finally, CT is the best technique to diagnose bronchopleural fistula, suspected with a persistent air leak in the pleural space for over 24 h.

## Foreign object aspiration

Foreign object aspiration (FOA) in children is a critical emergency that requires prompt recognition and intervention to prevent potentially life-threatening complications [41]. It commonly occurs in young children aged 1 to 3 years, with a peak incidence between 1 and 2 years old [41]. Common symptoms of FOA include sudden onset of coughing, choking, wheezing, dyspnoea, and in severe cases, respiratory distress and cyanosis [41]. If FOA goes clinically unnoticed, it may manifest later as recurrent pneumonia or asthma.

Imaging plays an important role, where CR is the initial imaging modality of choice in suspected FOA cases [42]. Typical radiological findings in FOA include unilateral hyperinflation due to a broncho-valve mechanism or atelectasis resulting from complete obstruction of the airways in the affected lung segment or lobe [42] (Fig. 7C). In case of post-obstructive pneumonia, the affected segment or lobe can also show consolidation. The bronchi and trachea should be scrutinised for a radiopaque foreign body. Signs of obstructive emphysema or mediastinal shift may also be present (Fig. 7C, F). If the medical history, clinical findings and radiograph are conclusive, cross-sectional imaging is not necessary and the child is transferred directly for treatment by bronchoscopy.

However, standard inspiratory CR might appear normal; therefore, expiratory CR should be performed to better detect air trapping and hyperinflation indicative of FOA. At times, a lateral decubitus radiograph of the chest may help, showing no lung collapse at the hyperinflated side. Alternatively, LUS or fluoroscopy can be used to observe pathological diaphragmatic movements, and LUS can depict unilaterally absent or reduced “lung sliding” sign, which may indicate hyperinflation of the affected lung [43].

CT is reserved for cases where CR is inconclusive, and when detailed anatomical information is required. CT can accurately localise the foreign body, assess its size and shape, evaluate associated complications (i.e., lung collapse and pneumonia) and decrease the rate of negative bronchoscopies [42, 44, 45] (Fig. 7F). MRI is seldom used in the acute setting of FOA due to logistical challenges and the need for sedation in young children. If cross-sectional imaging fails to locate the aspirated foreign object, due to the non-radiopaque nature of most foreign bodies, bronchoscopy is typically performed for both diagnostic and therapeutic purposes.

## Summary statement

Non-congenital pulmonary diseases encompass a broad spectrum of conditions affecting the respiratory system, with RDS being the most common and a primary concern in preterm neonates with surfactant deficiency. Chronicity of RDS results in BPD, the most common complication of extreme preterm birth, requiring chronic respiratory support. Conversely, TTN and MAS are common pulmonary diseases in term infants, with imaging aiding in diagnosis and differentiation. Congenital lung malformations like CPAM, BPS, CLO, BC, EDC and CDH are rare pulmonary diseases that present diagnostic and management challenges and where imaging plays a pivotal role in the diagnosis and long-term follow-up. Additionally, FOA demands prompt recognition and intervention, with chest radiography and computed tomography guiding diagnosis and treatment. Finally, pneumonia remains the most common pulmonary disease in children, where imaging is usually not needed to reach the diagnosis, but remains important for assessment of possible complications, such as empyema. Understanding the diverse presentations and imaging features of these conditions is crucial for effective management and improved outcomes in children.

## Patient summary

Multiple lung diseases can impact both premature and full-term infants, where imaging is crucial for accurate diagnosis and treatment guidance. Chest radiographs and lung ultrasounds are sufficient to diagnose most neonatal pulmonary disorders, including RDS, TTN, MAS, pneumonia, CDH, and FOA. Congenital lung malformations encompass a wide array of pulmonary conditions with varying symptoms. These malformations can either be asymptomatic or manifest with acute or recurrent symptoms from birth. Imaging is utilised to confirm abnormalities, often detected during pregnancy, and to monitor changes over time as the child grows. In symptomatic children, CT imaging is essential for assessing complications of pneumonia, possible recurrence of CDH and to guide bronchoscopy in FOA.

## Abbreviations

AP: Antero-posterior
BPD: Bronchopulmonary dysplasia
BPS: Bronchopulmonary sequestration
CDH: Congenital diaphragmatic hernia
CLM: Congenital lung malformation
CPAM: Congenital pulmonary airway malformation
CR: Chest radiograph
MAS: Meconium aspiration syndrome
PA: Posterior-anterior
RDS: Respiratory distress syndrome
TTN: Transient tachypnoea of the newborn
